# Periprosthetic Penile Abscess as an Unusual Cause of Refractory Urinary Tract Infection in an Elderly Patient With Diabetes: A Case Report

**DOI:** 10.7759/cureus.102082

**Published:** 2026-01-22

**Authors:** Ismael Palacio, George Michel, Kaira Portalatin, Kristal Arraut, Robert Hernandez, Adriana A Penate Armesto, Amir A Estil-las

**Affiliations:** 1 Internal Medicine, Larkin Community Hospital, South Miami, USA; 2 Medical School, University of Medicine and Health Sciences, Basseterre, KNA; 3 Internal Medicine/Infectious Disease, HCA Florida Kendall Hospital, Miami, USA; 4 Medical School, Ross University School of Medicine, Miramar, USA

**Keywords:** antibiotics, diabetes mellitus, esbl uti, extended-spectrum beta-lactamase bacteria, klebsiella pneumoniae, multidrug-resistant pathogen, penile abscess, periprosthetic penile abscess, refractory uti

## Abstract

A delayed periprosthetic abscess adjacent to a penile prosthesis, developing years after implantation, is a rare complication. We describe the case of an 81-year-old man with poorly controlled diabetes and post-stroke neurologic deficits, with a longstanding penile prosthesis, who developed an extended-spectrum beta-lactamase(ESBL) *Klebsiella pneumoniae* urinary infection and, subsequently, a peri-implant abscess. Despite empiric and targeted therapy, the patient deteriorated with systemic signs and progressive penile swelling until computed tomography revealed a deep periprosthetic collection. Because of the deep device focus, antibiotics alone were insufficient, prompting surgical removal of the indwelling device with debridement. This, combined with targeted therapy, led to resolution. This case underscores the need to maintain a high index of suspicion for periprosthetic infection in vulnerable elderly patients, particularly those with indwelling genitourinary devices, who present with recurrent or refractory urinary tract infections.

## Introduction

Urinary tract infections (UTIs) are one of the major causes of hospitalizations in the United States. Approximately 400,000 hospitalizations annually are attributed to a primary UTI [1]. While uncomplicated UTIs rarely require hospitalization, complicated UTIs, often associated with comorbidities, structural abnormalities, or resistant organisms, account for most UTI-related admissions. The hospitalization rate for complicated UTI is an estimated 626,000 admissions per year [2]. The incidence of these infections has been rising over the years, primarily due to an increase in antimicrobial resistance. This trend creates significant barriers to achieving optimal care [3].

*Escherichia coli* remains the predominant causative pathogen of UTIs; however, there has been a growing portion of infections caused by multidrug-resistant and healthcare-associated organisms such as *Klebsiella pneumoniae* and *Enterococcus faecalis* [4]. The rise in antimicrobial resistance, combined with the presence of comorbidities within a patient, has made UTIs more difficult to treat. Moreover, certain comorbidities may contribute to delayed diagnosis, significantly increasing the risk of infection progression. Conditions that lead to an immunocompromised state, such as diabetes, chronic neurologic diseases, immobility, prolonged catheter use, benign prostatic hyperplasia, the presence of chronically implanted foreign bodies like penile prostheses, and older age, place patients at greater risk of recurrent infections and higher complication rates [5].

Patients with poorly controlled diabetes mellitus are particularly susceptible to complicated UTIs due to impaired immune responses, poor glycemic control, delayed wound healing, and anatomical abnormalities associated with diabetic complications. Additionally, chronic neurological conditions such as cerebrovascular accidents, dementias, spinal cord injuries, and multiple sclerosis, among other conditions, further increase the risk of severe UTIs. This is often due to impaired autonomic control of the urinary tract, leading to urinary stasis or incomplete bladder emptying, reduced mobility, and frequent need for catheterization [6]. In such patients, infections may remain unrecognized until clinical deterioration or failure to respond to empiric treatment occurs.

Although genitourinary abscess formation is uncommon, the combination of immunosuppression, neurological dysfunction, and the presence of a foreign body prosthesis may significantly predispose patients to the development of such complications. Penile prosthesis infections are uncommon, with reported incidence rates of approximately 1-3% [7], typically occurring early after implantation; delayed periprosthetic abscess formation is rare.

This case highlights a rare presentation of a peri-implant penile abscess caused by extended-spectrum beta-lactamase (ESBL)-producing *K. pneumoniae* in a patient with poorly controlled diabetes and post-stroke neurologic deficits. It underscores how underlying metabolic and neurological conditions can delay the diagnosis and management of severe infections, emphasizing the challenges faced by vulnerable populations and the importance of prompt multidisciplinary interventions to improve patient outcomes. 

## Case presentation

An 81-year-old male residing in an assisted living facility was brought to the hospital with malodorous hematuria. He had a past medical history of type 2 diabetes mellitus, heart failure with reduced ejection fraction, hypertension, permanent atrial fibrillation on chronic anticoagulation with apixaban following a prior ischemic stroke, vascular dementia with right-sided hemiparesis, and a penile prosthesis implant.

On admission, the patient was disoriented and febrile with a temperature of 102°F. His cardiac rhythm revealed atrial fibrillation with a ventricular rate of 110 beats per minute. Physical examination demonstrated bilateral lower extremity pitting edema (grade 2). The initial genitourinary examination revealed a penis without deformity. There was no erythema, swelling, or tenderness on palpation. No palpable collection was identified along the penile shaft, scrotum, or around the scrotal pump. Due to his dementia, he was unable to report urinary irritative symptoms. Initial laboratory evaluation revealed leukocytosis, normocytic anemia, and hyperglycemia (blood glucose 362 mg/dL). Urinalysis demonstrated red and white blood cells and numerous bacteria. Hemoglobin A1c was 8.2%, consistent with poor glycemic control. Peripheral blood and urine cultures were obtained, and empiric antibiotic therapy with cefepime was initiated.

After 48 hours, the urine culture grew ESBL-producing *K. pneumoniae*. Cefepime was discontinued, and intravenous meropenem was initiated. The patient completed a five-day course of meropenem with resolution of sepsis and leukocytosis. Twenty-four hours later, he developed recurrent fever, leukocytosis, and tachycardia. A new, black, foul-smelling urethral discharge was observed, which was cultured. Local examination revealed penile swelling, erythema, warmth, and tenderness over the shaft, along with signs of urinary obstruction and bladder distension. A Foley catheter was inserted for urinary retention. The urethral discharge culture showed immature yeast forms only. Given the constellation of findings, recurrent sepsis, perineal inflammation, and prosthesis involvement, a complicated genitourinary infection was suspected.

**Figure 1:**
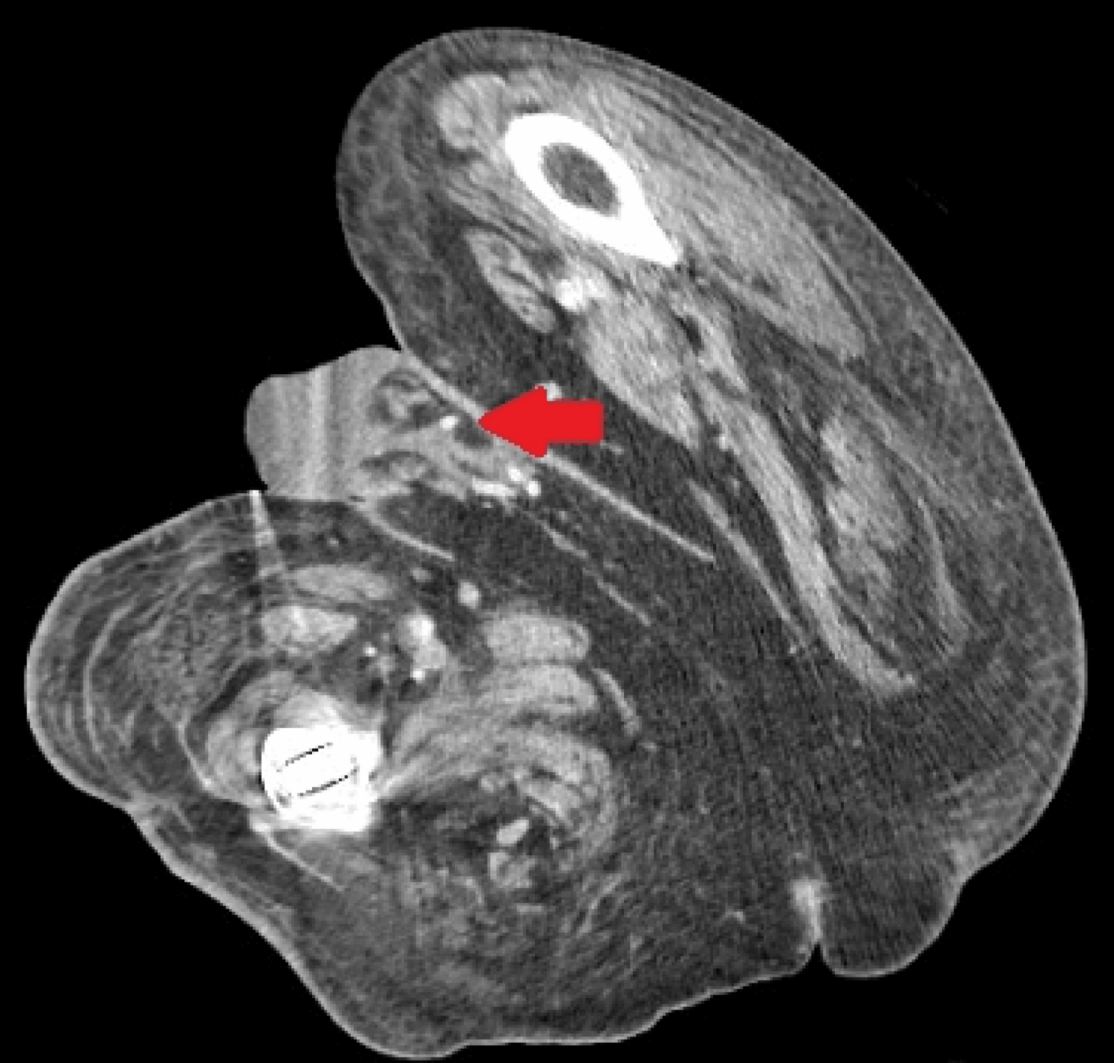
Contrast-enhanced CT (axial) of the pelvis at the level of the penis The red arrow points to the rim-enhancing fluid collection along the inferior aspect of the penile prosthetic cylinders with surrounding inflammatory changes.

**Figure 2:**
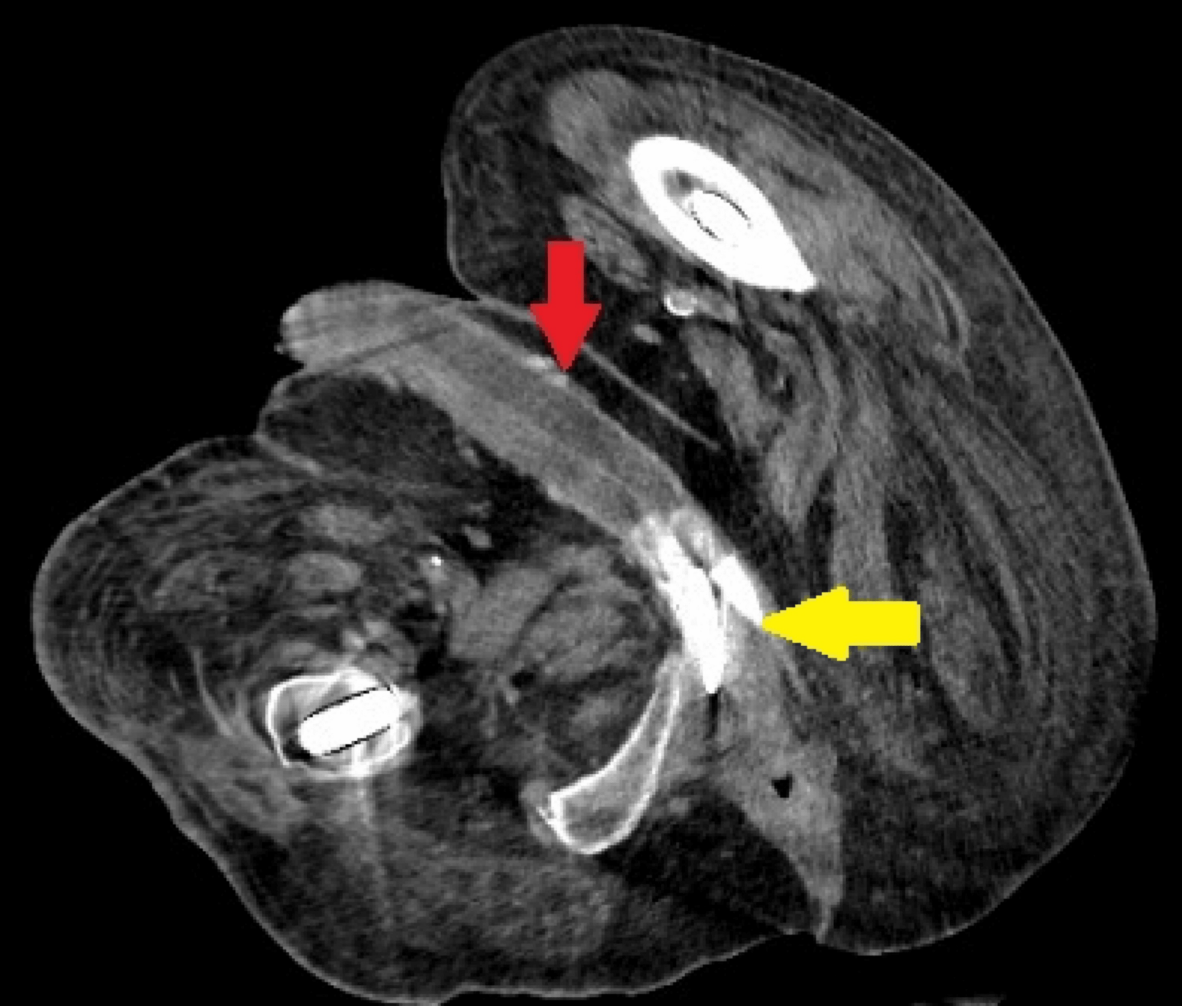
Contrast-enhanced CT scan (axial) of the pelvis The red arrow shows two intracavernosal cylinders surrounded by a small amount of low-attenuation fluid; the yellow arrow shows the two rear tip extenders of the penile prosthesis cylinder.

**Figure 3:**
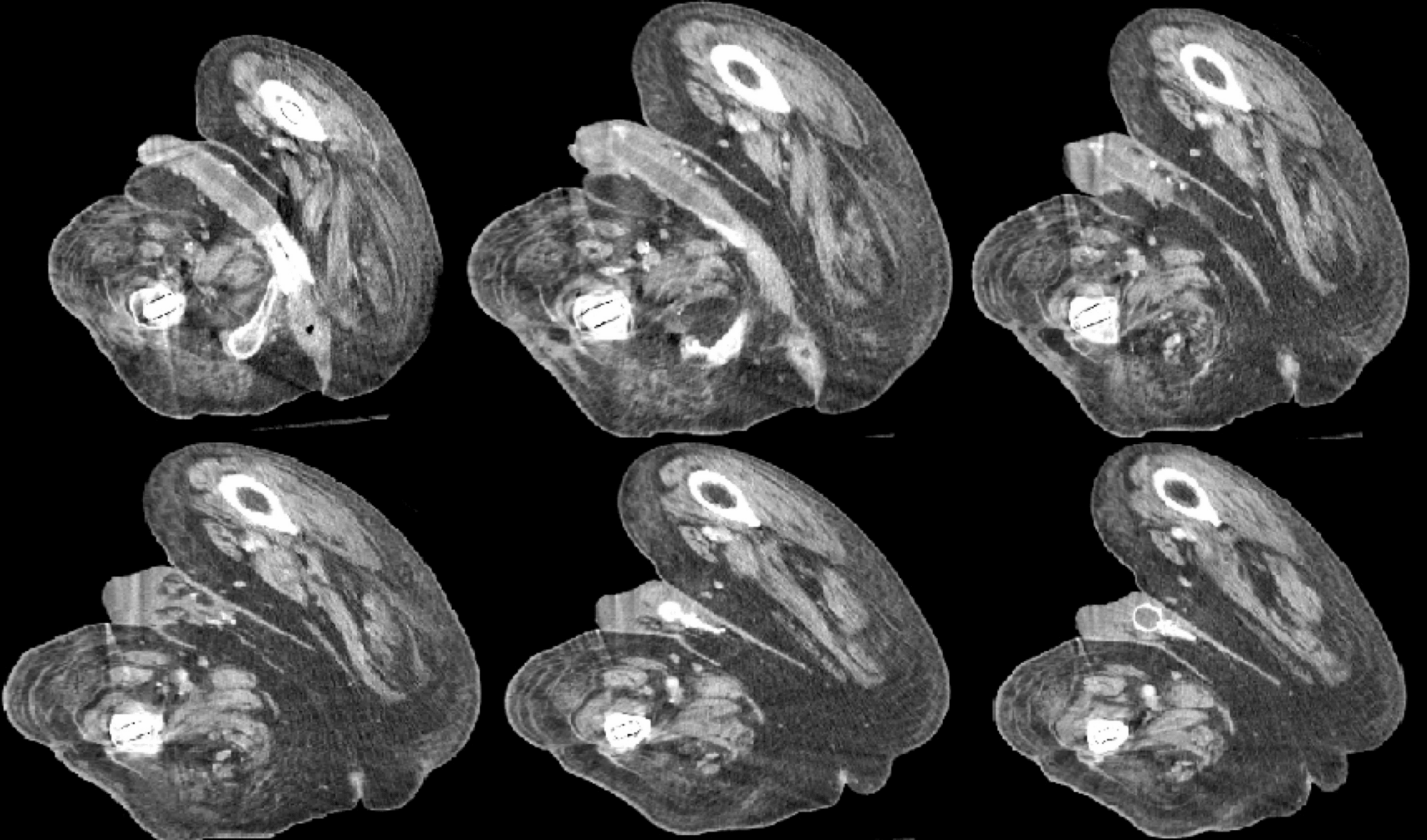
Serial contrast-enhanced CT scan (axial) images of the pelvis, progressing craniocaudally Bilateral intracavernosal penile prosthesis cylinders are seen with a small surrounding low-attenuation fluid collection and adjacent mild soft-tissue stranding, with beam-hardening artifact related to bilateral femoral implants.

A contrast-enhanced computed tomography scan of the abdomen and pelvis demonstrated moderate left hydroureteronephrosis with a transition point near the penile prosthesis reservoir and a periprosthetic fluid collection along the penile prosthetic cylinders, consistent with an abscess (Figures 1-3).

Due to concern for evolving Fournier gangrene, antimicrobial coverage was broadened to meropenem and vancomycin. Forty-eight hours later, sepsis parameters improved, and the urology team performed cystoscopy and complete removal of the penile prosthesis. The following day, the patient showed marked clinical improvement. The genital swelling decreased, though mild erythema persisted. Over the next 48 hours, inflammatory signs continued to resolve, and the Foley catheter was successfully removed without evidence of urinary retention.

Repeat blood and urine cultures were negative. Vancomycin was discontinued due to a drug-related rash, and linezolid was initiated for 10 days alongside meropenem, which was continued for a total of 14 days after source control. After completing intravenous therapy, the patient was transitioned to oral step-down therapy with fosfomycin 3 g every three days for two doses and doxycycline 100 mg every 12 hours for seven days to consolidate treatment and prevent recurrence.

## Discussion

Multidrug-resistant pathogens often challenge clinicians in both diagnosis and management, as they can present atypically, demonstrate limited response to standard antibiotic regimens, and lead to secondary complications. In patients with urologic prostheses and multiple comorbidities, these infections can seed the device, leading to unusual clinical outcomes. Although infection is a known complication following the surgical implantation of a foreign body device, the formation of an abscess adjacent to a penile prosthesis is a described but still uncommon manifestation. The incidence of primary penile prosthesis infection ranges from 1% to 3%. These infections are generally superficial or limited to the prosthetic device, typically presenting early after surgery [8]. Therefore, a periprosthetic abscess developing years after implantation is uncommon [9]. Studies have shown that such abscesses are usually associated with biofilm formation, bacterial seeding from urinary tract infections, or hematogenous spread [10,11]. In our patient, an ESBL-producing *K. pneumoniae* UTI, superimposed on poor glycemic control and neurologic deficits, provides a plausible mechanism for the development of the peri-implant abscess. 

Indwelling prosthetic devices, as well as diabetes and chronic neurologic conditions, are established risk factors for complicated UTIs. It is well known that diabetes is a leading noncommunicable disease and plays a significant role in the development of relapsing infections [12]. Several studies indicate that poor glycemic control (HbA1c > 7.5%), longstanding diabetes (over 10 years), and diabetic neuropathy increase the likelihood of severe infections, including emphysematous cystitis, pyelonephritis, and abscess formation, which may show limited response to broad-spectrum antimicrobial therapy [13,14]. This is attributable to hyperglycemia-driven immune dysfunction, which impairs host immune responses and reduces antimicrobial defenses [6]. Hyperglycemia also disrupts neutrophil chemotaxis, phagocytosis, and oxidative burst, thereby decreasing the effectiveness of bacterial clearance [15]. In addition, elevated urinary glucose concentration promotes a favorable environment for bacterial growth and enhances adhesion to uroepithelial cells [16]. When complications such as autonomic neuropathy are present, patients may develop neurogenic lower urinary tract dysfunction with impaired bladder sensation, incomplete emptying, and urinary stasis [6,17]. Diabetes has been identified as an independent risk factor for ESBL infection and is associated with higher prevalence and worse clinical outcomes [18,19].

Beyond diabetes-related complications, such as neuropathy, coexisting conditions affecting the nervous system or mobility further increase the risk of genitourinary complications. Studies have reported that the incidence of poststroke infections ranges from 25% to 65% [20]. This has been attributed, in part, to stroke-induced immunosuppression, in which multiple neuroendocrine and immune signals are activated, driving peripheral inflammation and lymphocyte apoptosis. Such immune dysregulation disrupts host homeostasis and diminishes antimicrobial defenses, increasing susceptibility to severe infection [16,21,22]. In addition, patients with post-stroke sequelae frequently exhibit anatomic and functional changes that impair neurologic control of the lower urinary tract, leading to incomplete bladder emptying, urinary retention, and urinary stasis, often necessitating indwelling urinary catheterization [16,23,24]. Moreover, post-stroke physical impairment and reduced mobility may compromise perineal hygiene, thereby complicating the clinical course. Although recent surveillance data suggest a decline in hospital-onset UTIs and catheter-associated UTIs, delayed recognition of deep-seated or periprosthetic infections continues to pose diagnostic challenges, particularly in elderly patients with multiple comorbidities and indwelling devices.

In our patient, this constellation of host and device factors, in the setting of an ESBL-producing *K. pneumoniae* UTI, provides a plausible source for seeding of the penile prosthesis and a delayed peri-implant abscess. Given this context, the ESBL phenotype of *K. pneumoniae* carries important therapeutic implications and is associated with recurrence, antimicrobial resistance, longer hospital stays, and worse clinical prognosis. ESBL-producing bacteria limit the activity of most β-lactams, narrowing empiric options and predisposing to treatment failure, as observed in our patient. Mechanisms of resistance to β-lactams include target site modification, reduced membrane permeability, and increased efflux pump activity. However, β-lactamase production, especially ESBLs and carbapenemases, remains the most common mechanism [12,25]. This may result from antibiotic overuse, biofilm formation, or genetic adaptations involving mutations and horizontal gene transfer [26]. Research has demonstrated that diabetes patients with ESBL-producing UTIs experience increased morbidity due to delays in initiating appropriate antibiotic therapy [12]. Consistent with this, our patient failed to improve with antibiotics, prompting imaging that identified a peri-implant abscess. Therefore, management should prioritize susceptibility-guided therapy together with urologic evaluation. Timely diagnosis, surgical device removal with debridement, and targeted therapy are crucial to prevent deterioration and recurrence and to achieve resolution. 

## Conclusions

This case highlights the importance of maintaining a high index of suspicion for periprosthetic infections in elderly patients presenting with recurrent or refractory UTIs, particularly those with indwelling genitourinary devices and diabetes mellitus. Early recognition of penile prosthesis-related abscesses can be challenging because initial findings may be subtle or absent, allowing the infection to progress silently until systemic involvement occurs. Imaging studies are crucial to identifying periprosthetic collections, while prompt surgical removal of the infected device remains the cornerstone of therapy.

Definitive source control, combined with targeted antimicrobial treatment and a carefully selected oral step-down regimen, is essential to achieve complete resolution and prevent recurrence. This case underscores how atypical presentations of device-associated infections can conceal severe underlying pathology and the critical role of timely diagnosis and decisive management in optimizing patient outcomes.
